# Supplementary material for: Development and Validation of Novel HPLC Methods for Quantitative Determination of Vitamin D3 in Tablet Dosage Form
Source: Pharmaceuticals (Basel). 2024 Apr 15;17(4):505. doi: 10.3390/ph17040505 (PMC11054345; doi:10.3390/ph17040505)
Supplement: Supplementary file 1 [file pharmaceuticals-17-00505-s001.zip › pharmaceuticals-2916312-supplementary.pdf]

**<Supplementary materials>**

**Development and Validation of Novel HPLC Methods for Quantitative Determination of Vitamin D3 in Tablet Dosage Form**

**Muhammad Saqib Gohar [1], Taj Ur Rahman [1], Ali Bahadur [2], Ashraf Ali [3,*], Sarah Alharthi [4,5] and Nora Hamad Al-Shaalan [6]**

**Table S1: System suitability of the developed method.**

| Replicates | Peak Areas |
|---|---|
| 1 | 67433 |
| 2 | 67286 |
| 3 | 67455 |
| 4 | 67238 |
| 5 | 67218 |
| 6 | 67173 |
| Mean | 67301 |
| Standard deviation | 117 |
| RSD | 0.1741 |

**Table S2: Precision repeatability of the developed method.**

| Replicate | Peak Area/Absorbance |
|---|---|
| Replicate 1 | 67433 |
| Replicate 2 | 67286 |
| Replicate 3 | 67455 |
| Replicate 4 | 67238 |
| Replicate 5 | 67218 |
| Replicate 6 | 67173 |
| Mean | 67301 |
| Standard Dev | 117 |
| Relative Standard dev (RSD) | 0.1741 |

**Table S3: Precision Ruggedness by different analyst variation of the developed method.**

| Analyst | Sample | Assay % | %RSD |
|---|---|---|---|
| 1 | 1 | 98.82 | 0.144 |
| | 2 | 98.56 | |
| | 3 | 98.60 | |
| 2 | 1 | 98.57 | 0.028 |
| | 2 | 98.54 | |
| | 3 | 98.60 | |

**Table S4: Robustness of the developed method.**

| Flow rate | Area | SD | %RSD |
|---|---|---|---|
| 2.4 | 68031 | 551 | 0.81 |
| 2.5 | 67206 | | |
| 2.6 | 68250 | | |
| **Mobile Phase composition** | **Area** | **SD** | **%RSD** |
| 82:18 | 66577 | 481 | 0.72 |
| 85:15 | 67173 | | |
| 87:13 | 67529 | | |

**Table S5: Limit of Detection LOD of the developed method.**

| Sample | Conc. % | Conc. mg/ml | Abs. | Standard Deviation | Slope | Conc. (ug/ml) at LOD | Absorbance at LOD |
|---|---|---|---|---|---|---|---|
| 1 | 80 | 0.00607 | 53707 | 117 | 7174221 | 0.0539 | 482 |
| 2 | 90 | 0.00675 | 60367 | | | | |
| 3 | 100 | 0.0075 | 67078 | | | | |
| 4 | 110 | 0.0082 | 73582 | | | | |
| 5 | 120 | 0.0098 | 80315 | | | | |

**Table S6: Limit of Quantification LOQ of the developed method.**

| Sample | Conc (%) | Conc. mg/ml | Abs. | Standard Deviation | Slope | Conc. (ug/ml) at LOQ | Absorbance at LOQ |
|---|---|---|---|---|---|---|---|
| 1 | 80 | 0.00607 | 53707 | 117 | 7174221 | 0.1633 | 1460 |
| 2 | 90 | 0.00675 | 60367 | | | | |
| 3 | 100 | 0.0075 | 67078 | | | | |
| 4 | 110 | 0.0082 | 73582 | | | | |
| 5 | 120 | 0.0098 | 80315 | | | | |

**Table S7: Specificity Placebo Interference in the developed method.**

| **Sample** | **Peak Area** |
|---|---|
| Aerosil USP | No peak is detected |
| Ossein mineral complex | No peak is detected |
| Primojel BP | No peak is detected |
| Mg Stearate BP | No peak is detected |
| Starch | No peak is detected |

**Table S8: Specificity blank Interference in the developed method**

| Diluent | Peak Area |
|---|---|
| Diluent and mobile phase | No peak is detected at the range of λ max 292 nm. |

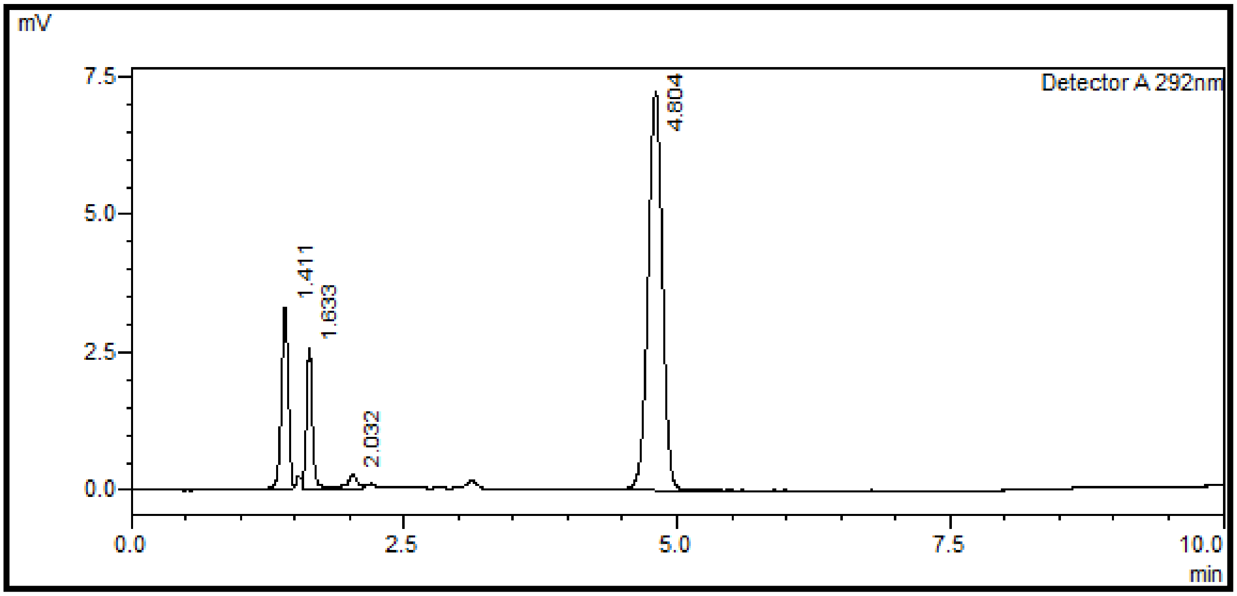


**Figure S1. Method precision (ruggedness) analyst variation**

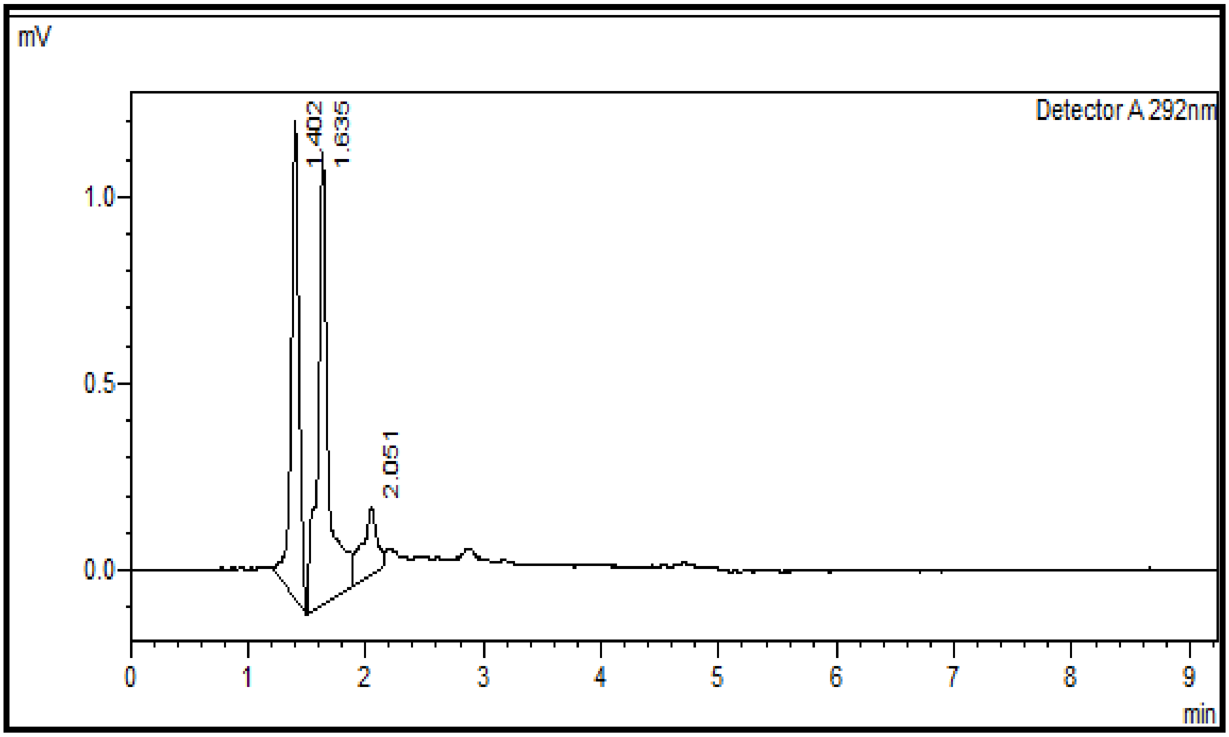


**Figure S2: Specificity placebo interference.**

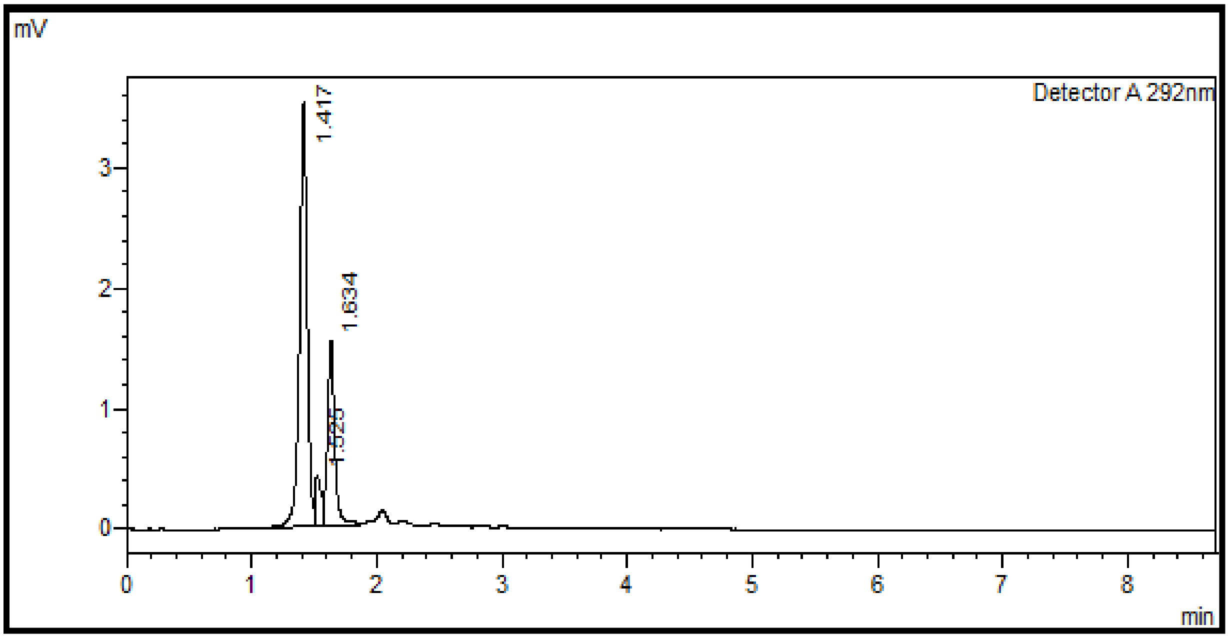


**Figure S3: Specificity blank interference-diluent.**

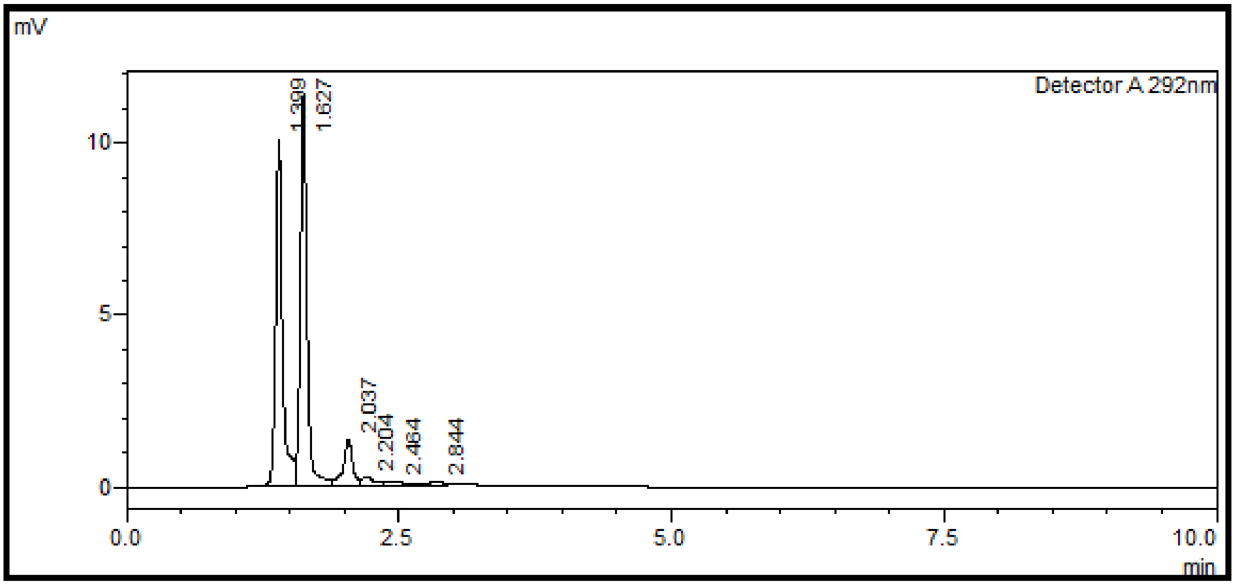


**Figure S4. Specificity blank interference-mobile phase**

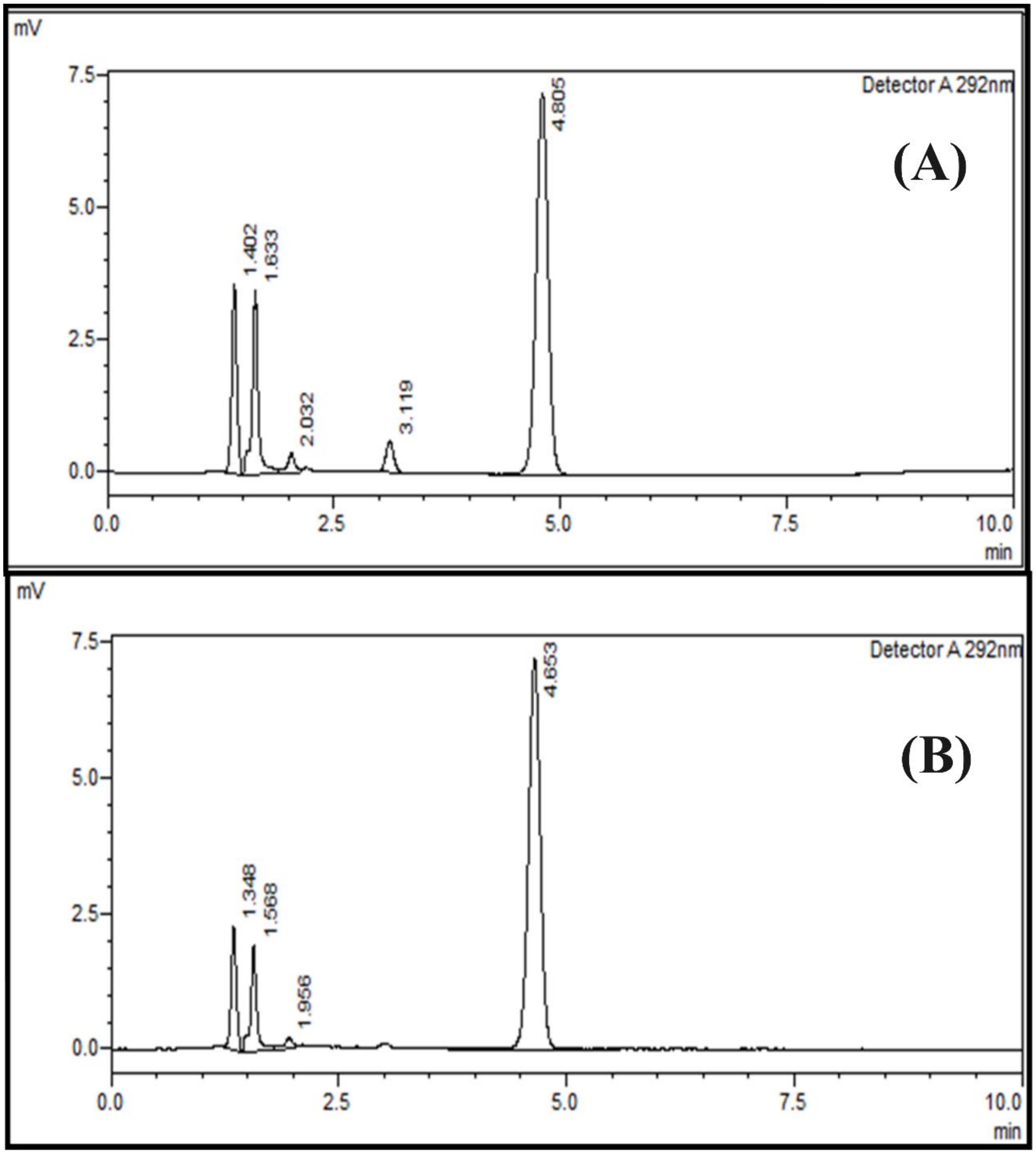


**Figure S5. Method precision (robustness) at the flow rates 2.0 ml/min (A) and 2.5 ml/min (B).**

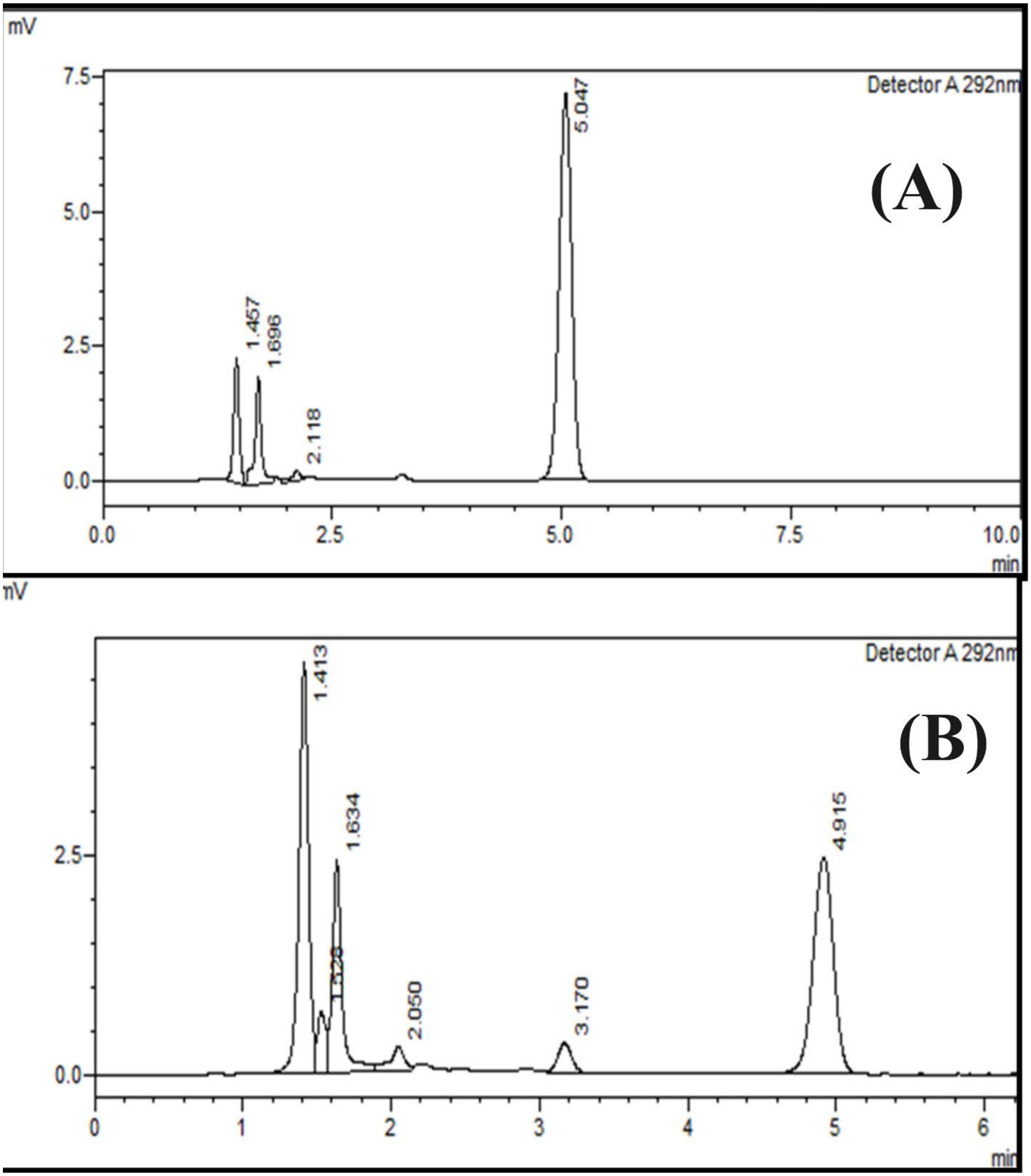


**Figure S6. Robustness of the method (mobile phase (80:20) (A) and (90:10) (B).**
